# Developing effective cancer vaccines: design and monitoring are critical

**DOI:** 10.1054/bjoc.2001.1839

**Published:** 2001-06

**Authors:** A Armstrong, S Dermime

**Affiliations:** CRC Department of Medical Oncology, University of Manchester, Paterson Institute for Cancer Research, Wilmslow Road, Manchester M20 4BX, UK


*British Journal of Cancer* (2001) **84**(11), 1433–1436
© 2001 Cancer Research Campaign
doi: 10.1054/ bjoc.2001.1839, available online at http://www.idealibrary.com on IDEAL®

http://www.bjcancer.com

Editorial

### Developing effective cancer vaccines: design and monitoring are critical

**A Armstrong and S Dermime**

CRC Department of Medical Oncology, University of Manchester, Paterson Institute for Cancer Research, Wilmslow Road, Manchester M20 4BX, UK

Published in this edition of the *BJC* are the results of a randomized phase II vaccine trial for patients with colorectal cancer. The trial, one of the largest to date, investigated the use of an anti-idiotypic antibody mimicking a tumour-associated antigen, in patients with advanced colorectal cancer. No survival advantage was demonstrated with the use of the vaccine. In an age where there is increased interest in the use of cancer vaccines it is important to assess the justification for their continued development.

### Vaccine immunology

Central to the renewed interest in cancer vaccine therapy is the increased understanding of mechanisms involved in an antigen-specific T-cell response, with animal studies demonstrating that although the humoral immune system may be relevant, it is cell-mediated immunity that is of critical importance in tumour protection (Golumbek et al, 1991; Dranoff et al, 1993). T cells recognize antigen only when displayed on the surface of the target cell or antigen-presenting cell as peptide fragments bound to the class I and II molecules of the major histocompatibility complex (MHC). MHC antigens are highly polymorphic, with different alleles binding different peptide epitopes derived from the tumour antigen. Class I MHC molecules, recognized by cytotoxic $CD8^+$ T cells, are present on the surface of virtually all nucleated cells. Class II molecules, recognized by helper $CD4^+$ T cells, are present on the surface of professional antigen-presenting cells such as dendritic cells. These highly specialized cells, which capture and process antigens released by tumour cell breakdown, are therefore able to present antigen to both cytotoxic and T-helper cells.

An effective T-cell response is now known to also be dependent on co-stimulatory molecules present at the time of antigen presentation. T-cell receptor engagement in the absence of co-stimulation results in T-cell tolerance.

### Limitations to the use of anti-idiotype vaccines

Anti-idiotypic antibodies raised against the unique antigen-binding site of an antibody may functionally mimic the antigen on the tumour-cell surface. Anti-idiotypic antibodies can therefore be used as surrogate tumour antigens for vaccine therapy. Where the anti-idiotypic antibody bears structural similarity to the tumour antigen anti-idiotypic antibodies should induce an antibody response to the idiotype and therefore to the tumour antigen itself. However, most B-cell epitopes will be to components of the anti-idiotype that do not mimic the tumour antigen.

Vaccine-based immunity is largely reliant upon the efficiency of the antigen-presenting cell that initially presents the antigen. Dendritic cells are the most effective antigen-presenting cells; one of the primary goals of vaccine therapy is to target these cells. Dendritic cells express Fc receptors that will bind anti-idiotypic antibodies, allowing the antigen to be presented in the context of class I and II MHC molecules, eliciting both cytotoxic and helper T-cell responses. Anti-idiotypic antibody treatment can, therefore, activate both arms of the immune system, in theory making them attractive candidates for cancer vaccines. It has been argued that the changed context of the epitope may facilitate the generation of an immune response, however, unlike native antigens, which contain several peptide epitopes capable of binding most MHC molecules, anti-idiotypic antibodies contain a limited subset of all potential peptide epitopes. They are therefore only likely to be effective in patients with a permissive MHC haplotype. When the tumour antigen that the idiotype mimics is known, computer-predicted binding motifs can be identified from the homologous regions, allowing clinicians to anticipate which patients may benefit from the vaccine. If this is not possible because the tumour antigen that the idiotype mimics has not yet been identified the idiotype may contains epitopes that bind only to rare MHC haplotypes and the vaccine will be ineffective for the majority of patients. Furthermore vaccines that contain only a limited number of T-cell epitopes may lack class I or class II motifs, potentially leading to a sub-optimal immune response. An additional concern with the use of minimal epitope vaccines is the increased potential for immune selection of tumours with subtle genetic variations that no longer express the peptide epitope(s). Immunohistochemical analysis of repeat biopsies from patients with metastatic melanoma who had initially responded to a peptide vaccine, but who relapsed despite the presence of peptide-specific cytotoxic T cells, revealed gradual loss of antigen expression in association with disease progression (Jager et al, 1997). Use of vaccines containing one or more whole antigens should induce a polyclonal immune response capable of recognizing multiple antigenic determinants and may prevent tumour escape.

### Genetically modified cancer vaccines: a superior approach?

The majority of tumour antigens are self antigens, to which there will be varying degrees of tolerance. Cancer vaccines must overcome this tolerance and encourage the immune system to see the antigen as foreign. It has been hypothesized in what is determined the danger model (Matzinger, 1994) that it is the context in which antigens are presented to the immune system that determines the outcome of antigen encounter. When an antigen is seen in the midst of inflammation and cellular damage that accompanies infection the outcome is typically activation. When an endogenously expressed antigen is encountered in the absence of these 'danger' signals, the outcome may be tolerance. Tumour cells that develop with little inflammation, at least in the early stages, result in tolerance. Cancer vaccines, unlike prophylactic vaccines against infectious diseases, must activate an immune response to an antigen to which it has already been exposed, and therefore need to be more immunogenic than traditional immunizations.

Advances in molecular biological techniques have allowed the development of vaccines that preclinical studies have demonstrated to be much more immunogenic than the early generation cancer vaccines. Molecular characterization of a variety of tumour antigens have provided the means for genes encoding these antigens to be configured as DNA, viral, or as genetically modified dendritic cell or tumour cell vaccines. Genetic vaccines, particularly viral-based systems, should allow more sustained antigen expression than protein or peptide vaccines.

One further advantage of molecular vaccines is the ability to incorporate genes encoding key elements of the immune response such as cytokines. The cytokine is produced in high concentrations at the site of the vaccine, altering the immunological environment and enhancing the activity of antigen-presenting cells and the activation of tumour-specific T cells. This is achieved without the side effects of systemic cytokine treatment. A phase I trial comparing GM-CSF gene transduced and non-transduced autologous renal cell vaccines provided preliminary evidence of the enhanced immunogenicity of the former by the induction of delayed type hypersensitivity responses against autologous tumour (Simons et al, 1997). Genes providing foreign helper T-cell epitopes such as tetanus toxoid can also be included, allowing more efficient priming of the immune response (King et al, 1998) and also providing an easy immunological readout for the success or otherwise of the clinical trial.

DNA vaccines are probably the simplest and most inexpensive genetic vaccine to generate. Having been shown to be effective in inducing anti-tumour immunity in several murine lymphoma (Hawkins et al, 1993; Stevenson et al, 1995; Syrengelas et al, 1996; King et al, 1998) and myeloma models (King et al, 1998), DNA vaccines are now entering clinical trials (Hawkins et al, 1997). Alternatives include recombinant viral vaccines that provide more efficient and reliable gene transfer and thus may be more potent vaccines than protein (Timmerman et al, 2001) or plasmid vaccines (own observations). One further advantage of the use of viral vectors is their intrinsic ability to initiate immune responses, with inflammatory responses occurring as a result of the viral infection creating the 'danger' signals necessary for immune activation. Expression of viral (and therefore foreign genes) should also act as an immunological adjuvant, further enhancing the immune response. Some viruses, including vaccinia viruses, are able to directly target antigen-presenting cells in vivo allowing for efficient presentation of incorporated antigens. Clinical trials of recombinant viral vectors are underway. A vaccinia viral vector encoding the E6 and E7 oncogenic proteins from HPV 16 and 17 was used to vaccinate patients with late-stage cervical cancer (Borysiewicz et al, 1996). The one patient that had a sustained clinical response to the vaccine also developed HPV-specific cytotoxic T cells.

Dendritic cells are key orchestrators of the immune response. Encouraging results with clinical trials involving dendritic cells, generated ex vivo, and loaded with tumour antigen prior to re-infusion (Hsu et al, 1996; Nestle et al, 1998; Kugler et al, 2000) have led to an interest in the use of genetically modified dendritic cells vaccines, with viral vectors currently the most efficient way to transduce dendritic cells (Arthur et al, 1997). One limitation to the use of viral vectors may be the level of pre-existing immunity to the vector. In murine models virally infected dendritic cell vaccines are more effective at inducing anti-tumour immunity in the presence of pre-existing anti-viral immunity than the recombinant viral vector alone (Brossart et al, 1997; Kaplan et al, 1999).

### Tumour burden and the success of vaccine therapy

The authors cite large tumour burden and advanced disease as one of the reasons for failure of the anti-idiotypic antibody trial. Certainly, it is becoming increasingly clear that as well as developing passive mechanisms for evading the immune response – such as antigen loss or MHC down-regulation – tumours themselves can actively inhibit the immune response. In a recent model mice vaccinated with irradiated tumour cells, genetically engineered to secrete GM-CSF and given simultaneously with a tumour challenge, were protected from that tumour challenge, with the protection mediated by T cells (Hsieh et al, 2000). Mice given the vaccine a week after tumour challenge were no longer protected, although the anti-tumor activity of the T cells could be restored following re-stimulation in vitro. It was demonstrated that the immunosuppressive factors IL-10 and TGF-beta, secreted by the tumour cells, inhibited T-cell function. It is likely that similar mechanisms exist in man, with one study finding raised IL-10 levels on 40 out of 99 patients with a range of solid tumours (Fortis et al, 1996).

### Evaluation of vaccine therapy: the importance of immunological endpoints

It is anticipated, then, that cancer vaccines will be more effective when given to patients with minimal levels of disease and may show no clinical effect in advanced disease. To justify the progression of a cancer vaccine through clinical trials (once initial studies have confirmed the safety of the approach), immunological endpoints are needed in order to improve the vaccine as necessary, and to optimize the vaccination schedule. It is possible that further optimization of the 105AD7 vaccine method could enhance the immune response and produce clear humoral or cytotoxic T-cell responses.

Difficulties in assessing the immune response to a vaccine hamper all vaccine trials. It is still not yet established which immune effectors are needed for the optimal anti-tumour immune response and therefore trials should examine different parameters, including both arms of the immune response. Enzyme-linked immunosorbent assays (ELISAs) reliably assess the humoral immune response induced by vaccination. Assessment of the cellular immune response is more difficult. Many assays require one or more rounds of in vitro stimulation of post-vaccination T cells, which potentially reduces the relevance of any result to what is actually happening in vivo. Assays such as the enzyme-linked immunospot (ELISpot), an interferon-$\gamma$-release assay which can detect the frequency of specific cytotoxic lymphocytes, are gaining popularity, but the relative importance of any positive result to clinical outcome remains unclear. The assay which currently appears in a number of trials to correlate most strongly

 © 2001 Cancer Research Campaign with a clinical response is delayed-type hypersensitivity (DTH) testing (Berd et al, 1990; Simons et al, 1997; Harris et al, 2000).

### Induction of autoimmune disease?

Clinical trials to date have demonstrated the safety of vaccine therapy for cancer. One consideration for vaccine trials is the choice of tumour antigen(s) incorporated in the vaccine with potentially the worst outcome of vaccinating against self-antigens being the induction of autoimmunity – it is well recognized that 20% of patients with melanoma that respond to IL-2 develop vitiligo. 105AD7 mimics CD55, a widely distributed complement regulatory protein that is over-expressed by a number of tumours. The therapeutic rationale thus depends on the ability of the immune response to distinguish the level of antigen expression. Indeed, cytotoxic T cells are able to identify tumour antigens that are over-expressed by tumour cells but are unable to recognize the same antigen on cells that express normal levels of the antigen. As an example of such a selective effect, vaccination of mice with murine p53 epitopes prevents the growth of tumours that contain high levels of the protein, without any damage to normal tissues (Mayordomo et al, 1996; Vierboom et al, 1997). There are, however, other mouse experiments where powerful vaccines induce strong and effective anti-tumor immune responses but at the expense of major autoimmune toxicity (Ludewig et al, 2000).

Thus if one proposed to use more powerful methods of vaccination to an antigen such as CD55 (which is expressed by many cells of the immune system), a degree of caution would be warranted because of the potentially disastrous consequences. Examples of other potential targets that are widely distributed include the Her2 growth factor receptor, which is over-expressed in many common tumours but also found in many normal tissues. Until the actual risk of autoimmunity is more quantifiable it may be prudent to initially vaccinate against tumour antigens whose expression is limited to less crucial cells.

### CONCLUSIONS

Currently there are no proven tumour vaccines and this is a further negative study. The trial is large, well conducted and there is no reason to doubt the result. Where do we go from here? Undoubtedly, vaccine design and antigen choice need careful consideration. Reliable and relevant immunological assays are needed as surrogate markers for the potential clinical benefit of any cancer vaccine, particularly as patients vaccinated in phase I/II trials are likely to have advanced disease. As more effective vaccines are developed the risk of autoimmunity will increase and must be carefully monitored and (where necessary) treated.

Nevertheless, there is great hope that cancer vaccines will offer a useful addition to more conventional oncological therapies. Preclinical studies are demonstrating the superiority of vaccines that take full advantage of recent advances in immunological understanding and molecular techniques over older vaccine formulations. It is anticipated that some of these advances will translate into successful clinical trials.

### ACKNOWLEDGEMENTS

Anne Armstrong and Said Dermime are supported by the Kay Kendall Leukaemia Fund.

### REFERENCES

Arthur JF, Butterfield LH, Roth MD, Bui LA, Kiertscher SM, Lau R, Dubinett S, Glaspy J, McBride WH and Economou JS (1997) A comparison of gene transfer methods in human dendritic cells. *Cancer Gene Ther* **4**: 17–25

Berd D, Maguire HC, Jr, McCue P and Mastrangelo MJ (1990) Treatment of metastatic melanoma with an autologous tumor-cell vaccine: clinical and immunologic results in 64 patients. *J Clin Oncol* **8**: 1858–1867

Borysiewicz LK, Fiander A, Nimako M, Man S, Wilkinson GW, Westmoreland D, Evans AS, Adams M, Stacey SN, Boursnell ME, Rutherford E, Hickling JK and Inglis SC (1996) A recombinant vaccinia virus encoding human papillomavirus types 16 and 18, E6 and E7 proteins as immunotherapy for cervical cancer [see comments]. *Lancet* **347**: 1523–1527

Brossart P, Goldrath AW, Butz EA, Martin S and Bevan MJ (1997) Virus-mediated delivery of antigenic epitopes into dendritic cells as a means to induce CTL. *J Immunol* **158**: 3270–3276

Dranoff G, Jaffee E, Lazenby A, Golumbek P, Levitsky H, Brose K, Jackson V, Hamada H, Pardoll D and Mulligan RC (1993) Vaccination with irradiated tumor cells engineered to secrete murine granulocyte-macrophage colony-stimulating factor stimulates potent, specific, and long-lasting anti-tumor immunity. *Proc Natl Acad Sci USA* **90**: 3539–3543

Fortis C, Foppoli M, Gianotti L, Galli L, Citterio G, Consogno G, Gentilini O and Braga M (1996) Increased interleukin-10 serum levels in patients with solid tumours. *Cancer Lett* **104**: 1–5

Golumbek PT, Lazenby AJ, Levitsky HI, Jaffee LM, Karasuyama H, Baker M and Pardoll DM (1991) Treatment of established renal cancer by tumor cells engineered to secrete interleukin-4. *Science* **254**: 713–716

Harris JE, Ryan L, Hoover HC, Jr, Stuart RK, Oken MM, Benson AB, 3rd, Mansour E, Haller DG, Manola J and Hanna MG, Jr (2000) Adjuvant active specific immunotherapy for stage II and III colon cancer with an autologous tumor cell vaccine: Eastern Cooperative Oncology Group Study E5283. *J Clin Oncol* **18**: 148–157

Hawkins RE, Winter G, Hamblin TJ, Stevenson FK and Russell SJ (1993) A genetic approach to idiotypic vaccination. *J Immunother* **14**: 273–278

Hawkins RE, Russell SJ, Marcus R, Ashworth LJ, Brissnik J, Zhang J, Winter G, Bleehen NM, Shaw MM, Williamson L, Ouwehand W, Stevenson F, Hamblin T, Oscier D, Zhu D, King C, Kumar S, Thompsett A and Stevenson GT (1997) A pilot study of idiotypic vaccination for follicular B-cell lymphoma using a genetic approach. CRC NO: 92/33. Protocol NO: PH1/027. *Hum Gene Ther* **8**: 1287–1299

Hsieh CL, Chen DS and Hwang LH (2000) Tumor-induced immunosuppression: a barrier to immunotherapy of large tumors by cytokine-secreting tumor vaccine. *Hum Gene Ther* **11**: 681–692

Hsu FJ, Benike C, Fagnoni F, Liles TM, Czerwinski D, Taidi B, Engleman EG and Levy R (1996) Vaccination of patients with B-cell lymphoma using autologous antigen-pulsed dendritic cells. *Nat Med* **2**: 52–58

Jager E, Ringhoffer M, Altmannsberger M, Arand M, Karbach J, Jager D, Oesch F and Knuth A (1997) Immunoselection in vivo: independent loss of MHC class I and melanocyte differentiation antigen expression in metastatic melanoma. *Int J Cancer* **71**: 142–147

Kaplan JM, Yu Q, Piraino ST, Pennington SE, Shankara S, Woodworth LA and Roberts BL (1999) Induction of antitumor immunity with dendritic cells transduced with adenovirus vector-encoding endogenous tumor-associated antigens. *J Immunol* **163**: 699–707

King CA, Spellerberg MB, Zhu D, Rice J, Sahota SS, Thompsett AR, Hamblin TJ, Radl J and Stevenson FK (1998) DNA vaccines with single-chain Fv fused to fragment C of tetanus toxin induce protective immunity against lymphoma and myeloma [see comments]. *Nat Med* **4**: 1281–1286

Kugler A, Stuhler G, Walden P, Zoller G, Zobywalski A, Brossart P, Trefzer U, Ullrich S, Muller CA, Becker V, Gross AJ, Hemmerlein B, Kanz L, Muller GA and Ringert RH (2000) Regression of human metastatic renal cell carcinoma after vaccination with tumor cell-dendritic cell hybrids. *Nat Med* **6**: 332–336

Ludewig B, Ochsenbein AF, Odermatt B, Paulin D, Hengartner H and Zinkernagel RM (2000) Immunotherapy with dendritic cells directed against tumor antigens shared with normal host cells results in severe autoimmune disease. *J Exp Med* **191**: 795–804

Matzinger P (1994) Tolerance, danger and the extended family. *Annu Rev Immunol* **12**: 991–1045

Mayordomo JI, Loftus DJ, Sakamoto H, De Cesare CM, Appasamy PM, Lotze MT, Storkus WJ, Appella E and DeLeo AB (1996) Therapy of murine tumors with p53 wild-type and mutant sequence peptide-based vaccines. *J Exp Med* **183**: 1357–1365

*© 2001 Cancer Research Campaign* 

Nestle FO, Alijagic S, Gilliet M, Sun Y, Grabbe S, Dummer R, Burg G and Schadendorf D (1998) Vaccination of melanoma patients with peptide- or tumor lysate-pulsed dendritic cells. *Nat Med* **4**: 328–332

Simons JW, Jaffee EM, Weber CE, Levitsky HI, Nelson WG, Carducci MA, Lazenby AJ, Cohen LK, Finn CC, Clift SM, Hauda KM, Beck LA, Leiferman KM, Owens AH, Jr, Piantadosi S, Dranoff G, Mulligan RC, Pardoll DM and Marshall FF (1997) Bioactivity of autologous irradiated renal cell carcinoma vaccines generated by ex vivo granulocyte-macrophage colony-stimulating factor gene transfer. *Cancer Res* **57**: 1537–1546

Stevenson FK, Zhu D, King CA, Ashworth LJ, Kumar S and Hawkins RE (1995) Idiotypic DNA vaccines against B-cell lymphoma. *Immunol Rev* **145**: 211–228

Syrengelas AD, Chen TT and Levy R (1996) DNA immunization induces protective immunity against B-cell lymphoma. *Nat Med* **2**: 1038–1041

Timmerman JM, Caspar CB, Lambert SL, Syrengelas AD and Levy R (2001) Idiotype-encoding recombinant adenovirus provide protective immunity against murine B-cell lymphomas. *Blood* **97**: 1370–1377

Vierboom MP, Nijman HW, Offringa R, van der Voort EI, van Hall T, van den Broek L, Fleuren GJ, Kenemans P, Kast WM and Melief CJ (1997) Tumor eradication by wild-type p53-specific cytotoxic T lymphocytes. *J Exp Med* **186**: 695–704

*© 2001 Cancer Research Campaign*

